# Electrochemical Lateral Flow Platforms: Pioneering the Future of Rapid Testing

**DOI:** 10.3390/molecules31081305

**Published:** 2026-04-17

**Authors:** Joao P. R. S. Carvalho, Isis C. Prado, Karyne Rangel, Jessica A. Waterman, Salvatore G. De-Simone

**Affiliations:** 1 Center for Technological Development in Health (CDTS)/National Institute of Science and Technology for Innovation in Neglected Population Diseases (INCT-IDPN), Oswaldo Cruz Foundation (FIOCRUZ), Rio de Janeiro 21040-900, Brazil; joaopedrorsc@gmail.com (J.P.R.S.C.); isis.prado@ioc.fiocruz.br (I.C.P.); karyne.rangelk@gmail.com (K.R.); jessicawaterman21@hotmail.com (J.A.W.); 2 Program of Post-Graduation on Science and Biotechnology, Department of Molecular and Cellular Biology, Biology Institute, Federal Fluminense University, Niterói 22040-036, Brazil; 3 Epidemiology and Molecular Systematics Laboratory (LEMS), Oswaldo Cruz Institute, Oswaldo Cruz Foundation (FIOCRUZ), Rio de Janeiro 21040-900, Brazil

**Keywords:** biosensor, lateral flow, electrochemistry, point of care

## Abstract

The increasing demand for advanced diagnostic technologies has positioned biosensor platforms as powerful alternatives to conventional analytical methods. Among them, lateral flow platforms (LFPs) are widely used for their speed, simplicity, and low cost. However, their limited sensitivity and lack of quantitative precision have spurred the development of enhanced systems incorporating electrochemical detection. Electrochemical biosensors offer significant advantages, including high sensitivity, excellent selectivity, and ease of miniaturization, which make them especially suitable for point-of-care testing (POCT). To address the limitations of traditional colorimetric LFPs, several strategies have been employed, such as the incorporation of nanomaterials, enzymatic amplification, and signal-enhancing labels. A particularly promising innovation is the direct integration of electrodes into LFPs, enabling real-time electrochemical readouts and enhanced analytical accuracy. Despite their potential, challenges persist, including manufacturing complexity, a lack of standardized protocols, and difficulties in scaling production for widespread adoption. Continued progress in developing hybrid platforms that combine lateral flow technology with electrochemical detection is crucial for expanding diagnostic applications in healthcare, environmental monitoring, and food safety. This work explores recent advances in electrochemical LFPs, reviewing current methodologies while discussing their advantages, limitations, and the future directions necessary to facilitate broader implementation and improve global diagnostic accessibility.

## 1. Introduction

In recent decades, the demand for rapid, accurate, and accessible analytical systems has grown significantly, driven by the urgent need for efficient diagnostics across various domains, including healthcare, environmental monitoring, and food safety. In this scenario, biosensors have emerged as cutting-edge detection systems, successfully overcoming the shortcomings of conventional techniques, including limited sensitivity, high costs, and reliance on elaborate infrastructure. These advanced devices integrate biological recognition components (e.g., enzymes, antibodies) with signal transducers that transform molecular interactions into quantifiable electrical outputs. This integration offers significant benefits, including enhanced selectivity, rapid response times, and the ability to miniaturize. In recent decades, the demand for fast, accurate, and accessible analytical systems has increased significantly, driven by the urgent need for efficient diagnostics across various domains, including healthcare, food safety, and environmental assessment. In this sense, biosensors have distinguished themselves as advanced detection platforms, effectively addressing the limitations of traditional methodologies, including low sensitivity, high costs, and the need for complex infrastructure. The versatility of biosensors allows their application in diverse environments, from advanced laboratories to remote locations with limited resources, making them ideal for point-of-care diagnostics [1,2].

Various well-established in vitro detection technologies are used by laboratories worldwide. However, the growing demand for on-site testing platforms is facilitated by the cost, infrastructure, and use of equipment in hard-to-reach areas. Recently, platforms related to low-cost, minimal, or no-equipment use, as well as simple assay formats, have gained prominence [3,4].

Among rapid diagnostic technologies, lateral flow assays (LFAs) stand out for their simplicity, economic cost, and usability, and are widely employed in the detection of biomarkers, pathogens, and contaminants. These devices function by capillary action, where a liquid sample migrates through a porous membrane, enabling specific biochemical reactions, such as antigen–antibody interactions, to occur, resulting in a visual signal, typically in the form of colored lines. However, despite their benefits, conventional LFAs face significant challenges, including insufficient sensitivity to detect analytes at low concentrations and difficulty in providing precise quantitative results. These limitations have motivated the search for innovative strategies to enhance the performance of these assays [5,6].

One of the most promising approaches to overcoming the limitations of LFAs is their integration with electrochemical biosensors, which offer high sensitivity, a wide detection range, and real-time quantification capabilities. Electrochemical biosensors are based on measuring currents, potentials, or impedances produced via redox reactions, enabling the detection of analytes at extremely low concentrations. The combination of these two technologies has opened new possibilities for developing hybrid platforms that merge the practicality of LFAs with the precision of electrochemical methods. These platforms are particularly relevant for medical applications, such as the early diagnosis of infectious diseases and the monitoring of inflammatory biomarkers, where speed and reliability are critical [7,8].

Furthermore, advancements in nanotechnology have played a fundamental role in enhancing electrochemical biosensors integrated with lateral flow platforms (LFPs). Nanoparticles composed of noble metals, such as gold and silver, and conductive nanomaterials have been utilized to strengthen electrochemical signals, improve conductivity, and increase the surface area of electrodes. These materials also allow functionalization with biomolecules, such as antibodies or aptamers, ensuring the specificity required to detect complex analytes in biological samples. Another significant innovation is the progress of label-free systems, which do not require chemical marker agents, simplifying the detection process and reducing costs. These advancements have driven the creation of more accessible and efficient devices capable of meeting global health demands [9,10].

The democratization of these technologies has the potential to reduce disparities in global health, particularly in low- and middle-income countries. Low-cost hybrid platforms may one day replace traditional methods, such as ELISA and PCR, which require expensive equipment and specialized labor. Thus, this review aims to explore recent advances in integrating electrochemical biosensors with the lateral flow platform, highlighting developed methodologies, their advantages, innovations, and challenges. Additionally, future perspectives for improving these technologies will be discussed, with a focus on process optimization, cost reduction, and expanding accessibility for public health, food hygiene, and environmental assessment applications. The combination of these two approaches promises to transform the landscape of rapid diagnostics, offering efficient and accessible solutions to global health and well-being challenges.

### 1.1. Immunochromatographic Assays

Lateral flow immunochromatographic assays (LFAs) are qualitative tests developed at the end of the 1960s and have applications in various fields, such as public health, helping to control and prevent the spread [11,12,13,14], food safety, protecting consumer health, and the presence of organisms or biological substances that can cause disease or harm to human, animal, or environmental health and scientific research [5,15,16]. Their wide acceptance, compared to other immunological methods, is due to several advantages, including rapid results without the need for specialized equipment, ease of execution, portability, and low costs [6,17,18]. Furthermore, these tests do not require sample preparation and are compatible with a wide variety of biological fluids, including whole blood, saliva, semen, urine, serum, or plasma [19,20]. The process of LFAs involves the migration of the sample along a membrane, where it encounters specific antibodies that bind to the target analyte, resulting in a visible signal. These assays are increasingly used in the emergency department (POCT) for timely and early diagnosis. One of the most popular and widely recognized examples of LFAs is the pregnancy test, which detects the hormone human chorionic gonadotropin (hCG) in urine. However, immunochromatographic assays have rapidly expanded to other applications, including the detection of infectious agents (such as SARS-CoV-2, HIV, *Mycobacterium tuberculosis*, etc.) and parasites (*Trypanosoma cruzi*, *Toxoplasma gondii*, *Plasmodium* sp., etc.), drugs of abuse, allergens, biological agents, and contaminated food [21,22,23,24].

Typically, lateral flow tests include various elements in their construction, such as the occurrence membrane, absorbent, and conjugation regions, as well as biological elements and buffers used. In this system, the analyte and markers run together to the test and control zones, where they are retained in specific areas, determining whether the analyte is captured or not. Although the overall sensitivity of these assays is reduced compared to other tests, studies have shown that the use of nanoparticles, such as gold and silver, and labels with fluorescence and magnetic properties can contribute to the sensitivity and accuracy of the test. Thus, with these modifications, it is possible to reduce the detection limit of the analyte present in the sample, improving the final quality of the assay [25,26,27].

Qualitative LFAs, although providing rapid results and being simple to handle, are used only for primary disease screening. Their results are based on the absence or presence of the desired analyte or marker, generating a visual outcome. However, there is a risk of false-positive or false-negative results due to technical errors or subjective interpretation. Given this scenario, the improvement of lateral flow test technologies is associated with quantitative assays, enabling concrete results about biomarker levels for disease diagnosis, such as the quantitative estimation of anti-Brucella IgG antibody levels in human plasma [6,18,28].

There are many types of readers for the quantitative measurement of LFAs. Colorimetric readers utilize particles with variable color intensity, like colored monodisperse latex or colloidal gold. In this type of test, the reader has mechanical parts capable of storing the LFA, a light source to illuminate both the test and control lines, a sensor to capture test images, and software to process the captured image and calculate the presence or concentration of the target analyte [29]. This type of reader is the most used due to its simplicity and small size. However, it has characteristics such as a high possibility of generating false-negative or false-positive results, as well as limited quantification and precision [30,31].

Fluorescence-based readers generally share most components with colorimetric readers, differing in the use of fluorescent markers that emit light at visible or infrared wavelengths. The system employs a wavelength-specific light source to induce fluorescence in the labeled particles. For this type of reader, multiplexing is possible, allowing the simultaneous detection of multiple analytes using fluorescent labeling particles with different wavelengths [29].

For magnetic LFA readers, the test result is obtained by measuring the intensity of the magnetic field, which can be based on monodisperse paramagnetic latex particles or supermagnetic iron oxide particles that use magnetic field sensors. Photometric readers, on the other hand, provide results by measuring the color generated by light-excited particles, while electrochemical readers are based on electrical properties such as voltage, current, and impedance. There are also dual-signal readers, which combine two or more of the reader types [32].

In addition to methodologies that utilize specialized readers, visual detection remains a key strength of the methodology. However, traditional methodologies, which rely primarily on colloidal gold nanoparticles (AuNPs), often suffer from low sensitivity, limiting their application to the detection of low-abundance analytes. To overcome this barrier, several signal amplification strategies have been developed to enhance biomolecule labeling, enabling clearer and more reliable visual reading without the need for complex electronic readers.

One strategy involves the use of polymeric nanoparticles in liposomes loaded with a high number of these dye molecules. While gold nanoparticles produce color through surface plasmon resonance, these carriers act as large capsules that concentrate thousands of dye molecules in a single spot, generating a more amplified visual signal. Antibody-functionalized liposomes can be bound to the test line, releasing more color molecules that accumulate and produce high contrast in the binding region, an effective principle for detecting very low concentrations of pathogens [33].

Another approach is enzyme-based catalytic amplification. In this method, antibody-conjugated nanoparticles exhibit catalytic activity, generating a colored product from a colorless substrate. One example is the use of gold and platinum bimetallic nanoparticles with peroxidase-like properties, which catalyze the reaction of substrates such as TMB (3,3′,5,5′-Tetramethylbenzidine), producing an intense blue precipitate in the test zone. This reaction amplifies the signal several times, improving the assay’s visual detection limit [34].

Another mechanism is based on a catalytic reduction reaction. The gold nanoparticles, already immobilized on the test line in the presence of the target analyte, act as catalytic nuclei for the reduction of silver ions (Ag^+^) to metallic silver (Ag^0^) from the added solution. This process results in the deposition of a dense layer of metallic silver on each of the AuNPs present. The visual effect is amplified, and the test line, which initially had a weak or barely visible signal to the naked eye, transforms into an intensely black or dark brown line, highly visible [35].

With the continuous advancement of nanotechnology, molecular biology, and the miniaturization of electronic devices, immunochromatographic assays are evolving toward more intelligent, sensitive, and integrated platforms. In the future, these devices are expected to perform multiplexed diagnostics in real-time, connected to the internet, with automated analysis based on artificial intelligence.

Such innovations will open new frontiers for personalized medicine, epidemiological surveillance, and real-time health monitoring. In a scenario of increasing demand for rapid and effective global health solutions, immunochromatographic tests will continue to be crucial for making diagnostic testing widely accessible.

### 1.2. Biosensors

Biosensors are analytical devices that integrate a biological recognition element with a physicochemical transducer to detect and quantify a sample of interest quickly, specifically, and sensitively. The primary characteristic that distinguishes them from other analytical methods is the combination of the selectivity provided by the biological component and the transducer’s ability to convert this recognition event into a measurable signal, which can be electrical, optical, thermal, or piezoelectric. This integration makes them versatile tools capable of providing real-time results, often without the need for extensive sample preparation, expanding their use in areas such as clinical diagnostics, environmental monitoring, food safety, and industrial applications.

The biological recognition element constitutes the central component of a biosensor, responsible for the interaction between the analyte and the sensor. Among the most commonly used are enzymes, which catalyze specific reactions and enable high sensitivity; antibodies, which provide high selectivity in immunological assays; nucleic acids and aptamers, which recognize sequences; Cells and tissues, which respond to more complex stimuli and are applied in toxicology and pharmacology, among others.

The second essential component is the transducer, responsible for converting molecular interactions into a detectable signal. Based on the physicochemical principle involved, biosensors can be classified into different types. Optical biosensors utilize fluorescence, absorbance, luminescence, or variations in refractive index, and are employed in applications that require high sensitivity. Piezoelectric biosensors detect mass changes in resonant crystals, enabling label-free, real-time analysis. Thermal biosensors measure variations in heat during biochemical reactions. Magnetic biosensors utilize magnetic nanoparticles as a detection method. Finally, the most widely used biosensor, electrochemical biosensors, rely on measurements of current, potential, conductance, or electrical impedance, and are widely used in portable and disposable devices [36,37,38].

Electrochemical biosensors are typically based on an enzymatic catalysis reaction that consumes or generates electrons. The enzymes used in this process are usually redox enzymes, which act as biocatalysts, accelerating reaction rates. The electrochemical reaction is measured using a three-electrode system, comprising a working electrode (where the reaction occurs), a counter electrode (to complete the circuit), and a reference electrode (to maintain potential stability). Both the analyte and the receptor are immobilized on the surface of the working electrode, which functions as the reaction transducer. The counter electrode serves as a current sink to complete the electrochemical circuit, allowing for the controlled application of current at the working electrode. Simultaneously, the reference electrode provides a stable potential reference point, ensuring the accurate measurement of the working electrode potential, which is essential for ensuring measurement accuracy, stability, and repeatability in electrochemistry [39,40].

**Figure 1:**
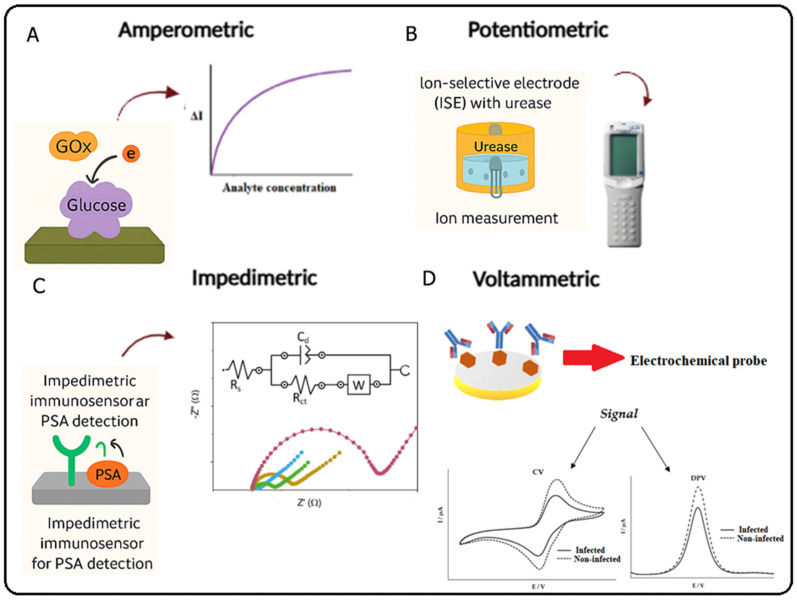
Schematic figure of types of electrochemical biosensors and their applications in diagnostics. (**A**) Increased current in relation to higher analyte concentration; (**B**) Ion-selective sensor in the presence of urease; (**C**) Increase in system resistivity after the presence of recognition between biomolecules; (**D**) Increased voltage signal after antibody coupling.

Electrochemical biosensors are classified into four types (Figure 1):

- Amperometry: Measures the electric current produced when a chemical substance oxidizes or reduces at an electrode in the presence of a biological catalyst (e.g., antibodies, enzymes, or microorganisms), maintaining a fixed potential.
- Potentiometric: Measures changes in electrical potential due to oxidation–reduction reactions, where the magnitude of the potential shift directly correlates with analyte concentration, enabling quantitative detection through this proportional relationship.
- Impedimetric: Measures electrical impedance, i.e., resistance or opposition to current flow, when a reaction occurs between the biomolecule and the analyte on a conductive surface.
- Voltametric: Measures the current resulting from a redox reaction when a variable voltage (potential difference) is applied to an electrode [41].

The earliest biosensor devices were glucose monitors that utilized enzyme-based detection principles to measure blood glucose concentrations. These pioneering systems established the foundation for modern biosensing technology. Since then, various types of biosensors have been developed, each tailored to a specific function and diagnostic purpose. These devices have the potential to serve as alternative tools for diagnostic testing for various diseases, as they can provide early, rapid, accurate, and sensitive detection. Electrochemical transduction offers simplicity and low cost, eliminating the need for additional signal conversion and enabling the progress of miniaturized systems for point-of-care applications. Currently, biosensors are used in various fields, including medical applications, agriculture, drug development, mobile devices, and Internet of Things (IoT) technologies that rely on biosensor technology [42,43,44,45].

**Figure 2:**
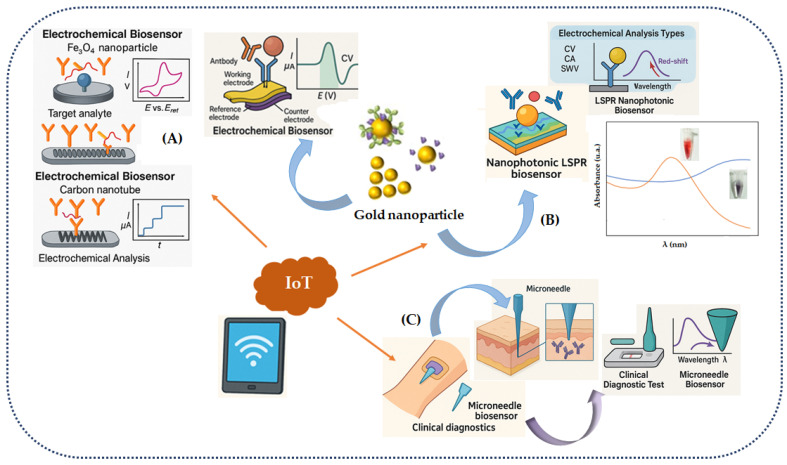
Diagram illustrating the interconnection between biosensors, nanotechnology, NM technology, and the IoT. (**A**) Simplified illustration of some nanomaterials described in the literature for making electrochemical biosensors; (**B**) Simplified illustration of the LSPR technique using gold nanoparticles; (**C**) NM biosensors.

Advances in nanotechnology have enhanced the sensitivity and precision of biosensors by utilizing various nanomaterials (Figure 2A). These are called nano-biosensors and currently constitute the majority of biosensors. Nanoengineered structures have demonstrated improvements, especially in sensor sensitivity and selectivity, due to their high surface-to-volume ratio, high reactivity, and unique physicochemical properties. Nanomaterials can detect analytes at trace-level concentrations, enabling significant advancements in the evaluation of individual molecules. These innovations are increasingly important and offer substantial improvements for biosensor development, with applications extending to areas of high social and environmental impact, such as biochemistry, clinical diagnostics, pharmacology, food hygiene, and environmental assessment [46,47,48].

In clinical diagnostics, the precise and sensitive detection of biomolecules plays an essential role in monitoring infectious diseases and preventing epidemics. Nanophotonic biosensors have risen to prominence as highly promising candidates for high-sensitivity miniature biosensors as a result of their ability to interact with light matter at the nanoscale for ultrasensitive biomolecular detection. The introduction of plasmonic biosensors, particularly those based on localized surface plasmon resonance (LSPR), has been one of the greatest advancements in ultrasensitive biomolecular detection (Figure 2B). These devices utilize metallic nanoparticles, such as AuNPs, which leverage collective electron fluctuations in the metal to detect changes in the refractive index of the surrounding environment, resulting in extremely high sensitivity. However, the advancement of miniaturized, cost-effective LSPR biosensors capable of operating directly at the point of care has faced significant challenges. The primary challenge lies in optical excitation and the requirement for precise coupling to collect high-quality spectral signals. To overcome these limitations, studies have explored the use of optical fibers combined with plasmonic nanostructures. Optical fibers have the advantage of being low-energy-loss waveguides, facilitating both light excitation and optical signal collection [49,50,51,52].

Nonetheless, altering the physical properties of these fibers to improve their plasmonic performance can compromise their mechanical characteristics, potentially leading to higher production costs. Yet, these obstacles can be overcome through manufacturing techniques that utilize additive manufacturing to position AuNP nanostructures at the ends of multimode optical fibers. This strategy, based on high-precision photoreduction, not only enables the production of LSPR biosensors on a small scale while maintaining high performance, but also ensures their versatility, favoring both large-scale manufacturing and application in disease diagnosis. Furthermore, additive manufacturing provides precise control over the size of nanostructures, ensuring high sensitivity in the resulting biosensors. Tests have shown that these devices can be easily functionalized with biomolecules, such as antibodies and nucleic acid receptors, enabling detection applications without the need for labeling, thereby opening up a wide range of potential applications. Thus, innovations in plasmonic optical fiber biosensor fabrication techniques open new possibilities for precision medicine, contributing to effective monitoring and rapid disease diagnosis, with the potential to improve responses to infectious outbreaks [53].

Another application of biosensors is the analysis of food contaminants, which pose a mounting challenge for global health. Developing sophisticated and reliable biosensing techniques with exceptional sensitivity, precision, and consistency is crucial for accurately detecting food contaminants and ensuring robust food safety monitoring. Biosensor development has emerged as a promising alternative for detecting specific agricultural pollutants. In this context, continuous efforts have been made to refine these detection systems, ensuring they are not only effective but also accessible and rapid, thereby promoting a more efficient response to the needs of the agricultural and food safety sectors. Various technologies have been integrated to overcome challenges associated with sample preparation, high costs, and the complexity of traditional analysis procedures. Among these innovations, approaches that combine enhanced sensitivity and ease of operation, such as electrochemical analysis methods (amperometric, impedimetric, and potentiometric), have proven effective in detecting pesticide residues in agricultural, water, and food samples, thereby reducing analysis time and detection limits. Similarly, fluorescence spectroscopy techniques using quantum dots have enabled significant advancements in constructing highly sensitive biosensors with detection limits as low as picomolar levels, allowing simultaneous detection of multiple pesticides in minutes [54,55].

A recent application of biosensors that has garnered interest among researchers worldwide is microneedle (MN) electronics, a promising technology that has been intensively studied. This methodology, known as smart electronics, integrates real-time monitoring of the target analyte with personalized therapy for treatment using microneedles (MNs) inserted into the patient’s skin (Figure 2C). Due to their semi-invasive structures, MNs enable efficient drug administration and target analyte detection without compromising tissue configuration [56]. Biosensors have applicability in the diagnostic area, as well as in various types of biosensors and their corresponding detection limits (Table 1).

#### Biosensors and Internet of Things

As technology progresses, the demand for convenience and ease of use has also increased. The IoT stands out as one of the most promising recent technological innovations, primarily due to its ability to reduce costs in multiple aspects, especially in healthcare systems. The system consists of a subset of computing that integrates interconnected sensors, microcontrollers, and transceivers to generate and provide detailed data to the user. Biosensors integrated with IoT technology enable continuous, real-time monitoring of biological and biochemical parameters, providing vast amounts of data for numerous applications, from healthcare to environmental monitoring. In healthcare, for example, this technology has enabled early disease detection, proving especially useful in preventive, therapeutic, and self-care applications. Within this realm, wearable devices, also known as wearables, have emerged as essential tools for facilitating more tailored patient treatments. These smart devices, which can be worn as accessories, tattoos, skin inserts, or fabrics, are internet-connected to collect, transmit, and receive data, enabling rapid decision-making IoT applied to biomedicine has become essential for supporting human care, allowing monitoring, management, detection, and automated responses based on clinical data reception, significantly contributing to cost containment in the sector [65,66,67].

Biosensors play a crucial role in this technological ecosystem, serving as portable, low-cost tools designed for rapid detection, providing alternatives to traditional analysis techniques that are often expensive and require specialized knowledge. Although the biosensor market exceeds $10 billion annually, its high cost remains a significant obstacle to widespread adoption, especially in low-income regions. Various initiatives are being developed to reduce these costs, including the reuse of biosensors. Sensor regeneration is a viable alternative to accelerate commercialization and enable large-scale applications, particularly in vulnerable contexts. In this transformative scenario, wearable devices have enabled substantial advancements by providing rapid responses, coupled with improvements in electronics and the development of low-power, high-speed mobile networks. The continuous growth of these devices has led to versions adapted for various uses, including handheld or body-worn devices, smart earphones, glasses, bracelets, and watches with medical and monitoring capabilities. Despite the variety of applications, the most relevant uses of wearables are concentrated in health tracking and continuous medical surveillance [68,69,70].

Within the technological ecosystem, electrochemical biosensors offer distinct advantages compared to other detection platforms, such as optical sensors. While optical techniques often rely on bulky and expensive equipment (such as spectrometers and specific light sources), increasing cost and limiting portability [7], electrochemical biosensors are based on the measurement of electrical currents or potentials, enabling extreme miniaturization and simplified integration with electronic circuits and microcontrollers [2]. This intrinsic characteristic makes them ideal for wearable and portable IoMT devices, as they consume significantly less energy, a critical requirement for battery-operated wearable gadgets, while offering real-time responses. Furthermore, the electrochemical platform offers high sensitivity and a low detection limit, often surpassing that of low-cost colorimetric tests, without compromising portability or ease of use [38]. The combination of low production cost, ease of miniaturization, low energy consumption, and robust analytical performance positions electrochemical biosensors not merely as an alternative to traditional laboratory techniques, but as the technologically superior option for direct and efficient integration into IoT networks, where autonomy, size, and connectivity are as crucial as analytical precision [2,38].

The advent of fifth-generation mobile networks (5G) marks a significant milestone in the evolution of wireless communications, offering large-scale connectivity, low latency, and high data transmission capacity, making it ideal for new applications in remote healthcare, medical robotics, and connected homes. This infrastructure supports the connection of billions of IoT devices, enabling the operation of thousands of devices and ensuring efficient support for systems with diverse data demands [71]. Thus, 5G technology becomes essential for strengthening the so-called Internet of Medical Things (IoMT), which integrates remote sensors, clinical wearable devices, and other technologies capable of tracking, recording, and transmitting medical data electronically, including information on treatment adherence, individual safety, physical activity, and vital signs [68].

Researchers have also extensively examined the integration of biosensors into food packaging as a key application of this IoT-enabled biosensing system. This approach aims to detect and identify early signs of food spoilage or quality maintenance. The combination of these biosensors in packaging with IoT can transmit information through smart networks, providing real-time data on food transportation and storage conditions. This integration enables continuous food care by providing access to the information needed for faster and more informed decision-making. The application of artificial intelligence in the biosensor-IoT combination can give substantial advances in food quality and integrity. This approach represents a technologically superior alternative to conventional food inspection methods, which are limited to evaluating physical parameters such as weight, volume, and visual appearance of products. This approach also considers the lack of need for human intervention to perform on-site inspections, which would increase the cost and time of analysis [72].

Lateral flow biosensors offer numerous advantages, including low-cost diagnostics, ease of handling, and detection, making them a highly efficient platform for diagnostics. Like microfluidics, they enable easy operation with small sample volumes, making them a highly efficient and accurate alternative. They also stand out for their speed, high throughput, and portability, making them a valuable tool for modern diagnostics. The convergence of IoT with microfluidics and lateral flow biosensors may signal significant progress in the field of biosensors. These analysis methods are defined as lab-on-a-chip platforms, characterized by portability, easy disposal, and real-time applicability. These systems can be easily coupled with a variety of transducers, including optical and electrochemical transducers, which will determine the detection method for each situation, thus expanding their versatility and diagnostic scope [73,74].

Smartphone technology transforms a simple communication device into a highly accessible platform. When combined with a small-scale microfluidic system, it provides a compact analytical method that requires only small sample volumes, making the platform more accessible. Integrating these devices into the IoT and the IoMT marks a significant advancement in digital health monitoring. The IoT offers a smart and effective solution for disease tracking and management, enabling continuous real-time monitoring and alerts. IoT-based devices can perform multiple functions, such as tracking disease spread, observing public health parameters, and supporting the implementation of preventive and therapeutic measures with greater precision. Such technological convergence results in a powerful and multifunctional tool for addressing complex epidemiological challenges [75].

Thus, biosensors have demonstrated their efficiency, effectiveness, and remarkable detection capabilities for diagnosing multiple compounds in biomedicine, agriculture, the food industry, safety, and water monitoring. Modernity has introduced these portable devices to enable patients to perform diagnostic tests independently, with the aid of a manual provided by the manufacturer. Nevertheless, a major challenge in biosensor applications lies in ensuring the device can effectively detect target signals and transduce them into usable outputs, such as electrical, electrochemical, acoustic, or optical signals. The transducer must be selective, sensitive to the analyte, fast, and have a low detection limit [42,76]. These characteristics can be better visualized by combining biosensors and nanomaterials to form multidimensional devices tailored to their specific applicability. This allows the construction of miniaturized devices with a high surface-to-volume ratio, good conductivity, and color-changing capability, among other improvements. In summary, by incorporating these features, any sensor can be optimized to serve as an ideal transduction platform for detection purposes [42,76].

## 2. Platform Construction

**Figure 3:**
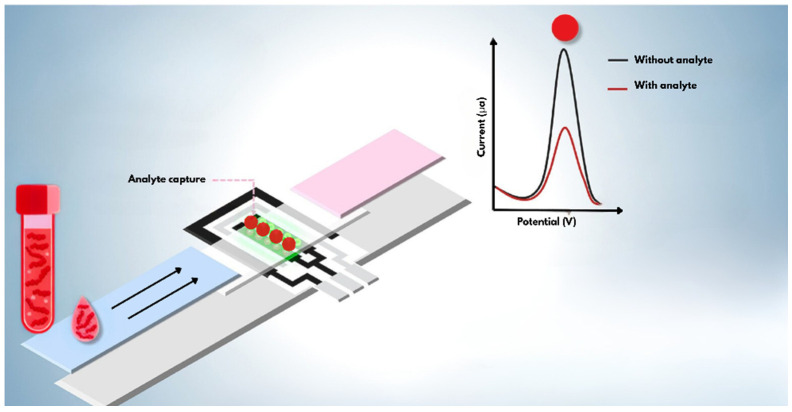
Schematic figure of the working principle of an electrode-integrated lateral flow system for analyte detection.

The innovative convergence of electrochemical biosensors and LFPs has emerged as a transformative technology in today’s point-of-care diagnostics landscape, representing a technological solution that can overcome the fundamental limitations of each approach when used in isolation. This strategic integration combines the exceptional analytical sensitivity, wide dynamic detection range, and precise quantification capabilities characteristic of electrochemical systems with the portability, operational simplicity, and low manufacturing cost that are hallmarks of conventional lateral flow tests. The result of this technological union is a new generation of hybrid diagnostic devices that redefine performance parameters for point-of-care applications, particularly in resource-limited settings and large-scale screening scenarios [77,78] (Figure 3).

The analytical superiority of this integrated approach becomes particularly evident when compared to traditional colorimetric systems, whose limitations in terms of sensitivity and quantitative capability have drastically restricted their diagnostic potential. Replacing conventional optical detection with electrochemical transduction mechanisms not only provides significant gains in detection limits, often associated with sensitivity parameters, but also enables the acquisition of real-time, quantitative data—a critical feature for applications requiring continuous monitoring or precise assessment of analyte concentrations. Additionally, the electrical nature of the signal generated in these hybrid systems substantially reduces interference common in optical-based methods, such as variations in sample turbidity, autofluorescence, or complex matrix effects, which often compromise the accuracy of results in conventional platforms [79,80].

From a technical implementation standpoint, the successful integration of these platforms requires innovative engineering solutions to multilayered challenges. The efficient miniaturization of electrochemical components for incorporation into the compact format characteristic of lateral flow strips represents one of the most significant obstacles, demanding parallel advances in various areas, including high-precision microfabrication techniques, development of optimized conductive materials, and standardization strategies for industrial-scale processes. The compatibility between the passive microfluidic systems of traditional lateral flow tests and the specific requirements of the electrochemical interface, particularly regarding electrical contact reproducibility and electrolytic environment stability, requires meticulous planning that balances analytical, manufacturing, and usability considerations [81,82].

The characteristic architecture of these hybrid systems preserves the fundamental principles of lateral flow operation but incorporates essential structural modifications to enable electrochemical functionality. The conventional detection zone is reconfigured to accommodate a strategically positioned microelectrode array, allowing efficient conversion of biomolecular recognition events into quantifiable electrical signals without compromising the operational simplicity that is a hallmark of the original platform. This advanced configuration enables direct and highly sensitive detection of specific biological interactions, such as antigen–antibody complex formation or nucleic acid hybridization, eliminating the need for auxiliary signal amplification steps that often add complexity to analytical protocols [83].

The analytical advantages provided by this integrated platform are particularly evident in applications requiring high performance in complex biological matrices. The electrochemical nature of the detection mechanism allows for the exclusion of optical interference, enabling reliable analyses in samples such as whole blood, urine, or saliva without the need for extensive pre-processing steps. Moreover, the multiplexing capability, enabled by the selective integration of multiple working electrodes functionalized with different receptors on the same platform, opens new possibilities for comprehensive diagnostic panels that can revolutionize screening and monitoring strategies [84].

Despite these promising advancements, significant challenges remain on the path to consolidating this technology as a standard in decentralized diagnostics. The long-term stability of active biological elements immobilized on electrode surfaces under varying storage conditions remains an active area of investigation, requiring the development of new preservation and encapsulation strategies. Equally important is the challenge of industrial scalability, which demands standardization between the high-precision fabrication processes required for electrochemical components and the mass-production techniques characteristic of lateral flow test manufacturing. Comprehensive clinical validation in diverse epidemiological scenarios and under varying operational conditions represents another essential milestone for transitioning this technology from the laboratory environment to routine clinical applications [85,86].

Prospects for this hybrid technological platform are particularly exciting when considering its potential integration with other emerging innovations. Coupling with low-energy wireless communication systems could enable automated transmission of diagnostic data to digital health platforms, facilitating real-time population monitoring and integration with early warning systems. The incorporation of artificial intelligence algorithms for advanced electrochemical signal analysis could provide not only greater diagnostic accuracy but also the identification of subtle patterns that could inform personalized therapeutic strategies. The continued development of advanced nanomaterials with optimized electrocatalytic properties promises to further elevate analytical performance parameters, while innovative surface functionalization approaches could significantly expand the range of detectable biomarkers [87,88].

The integration between electrochemical platforms and lateral flow systems is crucial to ensure the reproducibility, reliability, and consistent performance of analytical devices, particularly in applications such as environmental monitoring, point-of-care diagnostics, and industrial quality control. To achieve these objectives, scientific literature proposes various construction and optimization methodologies, including the functionalization of electrode surfaces with nanomaterials, the engineering of porous membranes for flow control, and the integration of these systems with microfluidic devices. These approaches aim to enhance analytical sensitivity, minimize interference, and ensure accurate results, thereby making the devices more robust and suitable for both field and laboratory applications.

### 2.1. Enzyme-Based Systems

Enzymes are well-known for their individual properties. One of their most significant advantages is their ability to amplify signals by catalyzing chemical reactions that produce electrochemically active products, such as ions or molecules, that alter the measured current or potential, enabling the detection of compounds at extremely low concentrations. Additionally, enzymes demonstrate remarkable specificity, selectively recognizing and interacting with their substrates, which reduces interference from other substances. Another key benefit is their ability to bind with biomolecules, such as antibodies or nanoparticles, facilitating targeted applications for analyses in biological fluids, including blood, saliva, and urine.

Platforms utilizing these systems generally establish direct contact between electrodes and NM or cellulose substrates, allowing real-time measurement of reagents as they migrate through the device. For example, this study proposed the development of an electrochemical lateral flow sensor (eLFIA)-based assay for detecting two inflammatory biomarkers: interleukin-6 (IL-6) and C-reactive protein (CRP). The methodology involved fabricating a three-electrode system (working, counter, and reference) directly onto a nitrocellulose membrane by screen printing. Carbon ink was used for the working and counter electrodes, and Ag/AgCl ink was used for the reference electrode. This integration eliminated the need for external electrodes, reducing costs and increasing system stability. The detection methodology was based on an enzymatic amplification strategy using horseradish peroxidase (HRP), which catalyzes the oxidation of tetramethylbenzidine (TMB) to form an electroactive precipitate (TMB_ox_) on the surface of the working electrode. Quantification was performed by SWV. The sensor demonstrated a very low limit of detection (LOD) of 0.17 pg/mL for IL-6 and 0.54 pg/mL for CRP, with high sensitivity and a linear response in the ranges of 0.5–50 pg/mL and 1–125 pg/mL, respectively. The selectivity of the test demonstrated a minimal response to other interferents, such as IL-10 and TNF-α, and the entire detection process was completed within 20 min.

Some of the main strengths of this study include innovative electrode integration, which reduces costs, the high sensitivity, and the portability of the system, which utilizes a smartphone. However, the “hook” effect at high concentrations (above 50 pg/mL for IL-6) requires careful consideration of optimal dilution. Furthermore, the complexity of electrode fabrication and stability studies poses a significant challenge, drastically affecting the potential for mass production of this system [89].

In another study, ferrocene was employed as a marker associated with a specific antibody targeting the ACE-2 region, which was immobilized on NM above the working electrode. This setup allowed the marker to participate in redox reactions, thereby indicating the presence of the target protein [78]. Similarly, a lateral flow electrochemical device was developed for the quantitative detection of C-reactive protein (CRP) in saliva. The methodology was based on the immobilization of specific antibodies onto a glass fiber membrane connected to an NM, integrated into an electrochemical system featuring carbon electrodes. Detection of the protein is achieved by measuring the current generated during the antigen–antibody interaction, facilitated by antibodies conjugated with alkaline phosphatase. This enzyme oxidizes the substrate 2-phospho-L-ascorbic acid, thereby amplifying the signal. The process is further optimized to ensure high sensitivity and reproducibility using CRP standards under defined conditions. The analysis is completed within minutes, and quantitative results are obtained directly from the device [90].

In another study, an electrochemical immunoassay for the ultrasensitive detection of biomarkers in clinical samples was proposed, with potential applications in point-of-care diagnostics. The method employs a signal amplification strategy based on electrochemical redox cycling mediated by β-galactosidase, which oxidizes a specific substrate and enables the detection of 4-amino-1-naphthol at a defined pH. This approach ensures rapid and selective interaction, minimizing the background noise, and eliminating the need for additional processing steps [91].

In literature, a test designed to detect prostate-specific antigen (PSA) positioned the electrode beneath the zone where the specific antibody is captured. This setup employed urea as a signal generator; the enzymatic hydrolysis of a urea-based substrate induced changes in the system’s capacitive signals, enabling detection [89].

### 2.2. Nanoparticle-Based Systems

Metallic nanoparticles are frequently used in lateral flow systems linked to electrochemical devices due to their unique characteristics, like high electrical conductivity, which enables the efficient amplification of electrochemical signals crucial for quantifying biomarkers at low concentrations. Their ability to functionalize with biomolecules, such as antibodies or enzymes, allows specific capture of the target, in addition to their stability and biocompatibility.

One study employed an innovative methodological approach that combined lateral flow immunochromatography with electrochemical detection using anodic stripping voltammetry (ASV). Bismuth ions (Bi^3+^) were used as metal markers. The ions were coupled to the anti-HCG antibody using a bifunctional chelating agent called diethylenetriamine pentaacetic acid (DTPA). Immediately after the immunochemical reaction, the ions were released by acidification and quantified by ASV on a disposable screen-printed electrode integrated directly into the strip’s nitrocellulose membrane. This methodology enabled the development of an electrochemical chromatographic immunosensor (EBI) for the detection of human chorionic gonadotropin (HCG) in serum samples.

The sensor demonstrated a limit of detection (LOD) of 1 mIU of HCG, characterized by high sensitivity and selectivity, which is attributed to the specificity of the immunological reaction and the selective detection of bismuth. With a total test time of 10 min, validation was performed using serum samples, which showed different responses for concentrations of 25 and 50 mIU of hCG, without interference from matrix components or other elements.

As is characteristic of the technique, the high sensitivity of ASV detection, which overcomes the limitations of optical interference of conventional methods, the operational simplicity of the integrated system, and the low cost associated with disposable electrodes, as well as the possibility of using markers for multiplexed detection, are strengths. However, the need for complex conjugate between DTPA and the antibody, the limited stability of the stored conjugate (4 °C), and the use of mercury in the formation of the Hg film on the electrode for detection generate several problems, mainly associated with environmental issues, necessitating optimizations to transform the product [92].

Another extensively studied nanoparticle is gold, which has been widely used due to its broad applicability. In one approach, it served as a signal amplifier for alpha-fetoprotein (AFP) detection in a sandwich-type immunoassay. Anti-AFP antibodies conjugated with horseradish peroxidase (HRP) enzymes were incorporated into gold nanoparticles. Following migration through the membrane, detection was achieved by oxidizing O-phenylenediamine (OPD) in the presence of H_2_O_2_, generating a peak in the electrochemical signal via square wave voltammetry [93].

Lin et al. used CdSe@ZnS quantum dots functionalized with secondary antibodies as nanoprobes for PSA detection. Following the operation of the immunochromatographic system and antigen capture in the region above the electrode, a hydrophobic barrier formed within the interaction zone. Upon the addition of HCl, cadmium ions were released, and SWV was employed to detect the antigen [92].

For the detection of myeloperoxidase (MPO), the study employed magnetic nanoparticles labeled with antibodies. These nanoparticles reacted with the analyte and specific enzymatic markers in solution. Following the interaction, they were deposited onto an integrated electrochemical lateral flow platform. After a washing step, the oxidized substrate produced detectable signals on the electrode surface [94].

The study also presents the development of an electrochemical lateral flow sensor. The method uses a cellulose strip with a conjugate pad containing gold nanoparticles (AuNPs) functionalized with ferrocene. These nanoparticles bind to the dengue NS1 antigen via specific antibodies. As the immunological reaction occurs along the strip, the resulting antigen–antibody complex migrates to a capture zone, forming a sandwich-type structure. Detection is carried out electrochemically, with the ferrocene redox marker embedded in the nanoparticle, generating a signal proportional to the concentration of NS1 protein. This approach demonstrates high sensitivity and ease of prototype assembly, which are its main advantages [95].

### 2.3. Label-Free Systems

Label-free electrochemical detection techniques are promising tools for evaluating biomolecules such as proteins, nucleic acids, and antibodies, as they eliminate the need for chemical or biological markers (Table 2). These techniques rely on detecting changes in the electrochemical properties of a functionalized surface (such as variations in current, potential, or impedance) caused by the specific interaction between the analyte and a recognition element immobilized on the electrode. The main advantages of label-free strategies include their simplicity and speed, as they bypass steps like nanoparticle or molecular labeling, thereby reducing costs, assay time, and sample preparation requirements [92].

The research presents an approach for diagnosing urinary tract infections (UTIs) using an immunochromatographic electrochemical biosensor. The methodology focused on the development of a label-free platform that uses Electrochemical Impedance Spectroscopy (EIS) to quantify Prostaglandin E2 (PGE2) in urine samples. The sensor was constructed using a system of gold microelectrodes deposited directly onto a nanoporous membrane (Cytiva, Marlborough, MA, USA.), which served as a support for immobilizing biomolecules and the lateral flow matrix. The analyte is captured by a monoclonal antibody specific to PGE2, which is present on the electrodes through the crosslinker DSP (dithio-bis-succinimidyl propionate), generating stable bonds with the gold matrix.

With a detection limit of 100 pg/mL and a wide dynamic range of 100 to 4000 pg/mL, the approach encompasses the “normal” physiological variation in human urine. Sensitivity was enhanced by the confinement generated by the porous membrane structure, which increases the surface area and antigen–antibody binding kinetics. Specificity was ensured by the selectivity of the biomolecules and the electrochemical reading methodology, which minimizes electrostatic interference from the urinary matrix, enabling results to be presented in 5 min. Furthermore, validation in artificial and human urine demonstrated acceptable reproducibility (CV% < 20%) in accordance with clinical guidelines.

The elimination of sample preparation steps, the low volume required (<100 µL), and rapid reading, combined with the absence of the need for enzymatic or redox markers, reduces complexity and cost. However, limitations remain, such as sensitivity to variations in urinary pH and dependence on stable antibodies. Scalability is associated with the main need for more comprehensive studies with diverse samples to consolidate the correlation between PGE2 levels and UTI diagnosis in the sample [96].

Another study describes the development of a portable, paper-based electrochemical biosensor for the rapid and accurate diagnosis of COVID-19. Designed to complement traditional methods such as RT-PCR, the sensor detects specific antibodies produced in response to SARS-CoV-2 infection. A cellulose surface coated with gold nanoparticles (AuNPs) forms the electroactive region. Upon antibody binding, the system detects changes in the conductivity of a redox-active species in the surrounding solution, in the sample [97].

A related work, also targeting the same disease, introduces an innovative approach for developing an electrochemical aptasensor for SARS-CoV-2 detection. The platform utilizes a specific aptamer targeting the viral spike protein, which is immobilized on a polyester substrate coated with a gold electroactive layer. Detection is carried out using electrochemical impedance spectroscopy, a label-free technique that monitors changes in electrical impedance at the electrode interface upon aptamer-antigen binding [98].

This method entails the development of a vertical-flow paper immunosensor, where antibodies targeting the Influenza H1N1 virus are anchored onto gold electrodes patterned on NMs. Detection is carried out using two complementary techniques: electrochemical impedance spectroscopy (EIS) and colorimetric analysis. The system incorporates a dual-porosity sample pad, featuring 11 µm pores in the upper layer and 0.45 µm pores in the lower layer, which acts as a selective filter, allowing viral particles to pass through while retaining larger contaminants. When viral antigens in the sample interact with horseradish peroxidase (HRP)-labeled antibodies, immunocomplexes are formed and migrate toward the gold electrode, where variations in charge transfer resistance (R_CT) are measured by EIS. Concurrently, unbound antibodies are captured in a separate detection zone, producing a colorimetric signal proportional to the antigen concentration. Both approaches offer highly sensitive detection, achieving limits as low as 3.3 PFU/mL with EIS and 1.34 PFU/mL with colorimetry, all without the need for external reagents, resulting in a portable, affordable, and rapid diagnostic tool for respiratory infections [99].

**Table 2:** Performance comparison of lateral flow electrochemical biosensors for various biomarkers.

Diagnostic	Analyte	Transduction Technique	LOD	Detection Range	Time to Result	Surface Type	Ref.
Prostate cancer	PSA	Impedimetric	10 ng/mL	0–30 ng/mL	>20 min	CA/NC	[100]
COVID-19	SARS-CoV-2	DPV	2.98 pg/mL	0.01 to 500 ng/mL	12 min	Graphene/NC	[78]
Inflammation	C-reactive protein	Chronoamperometry	3 ng/mL	Not reported	15 min	Gold/NC	[90]
Myocardial infarction	Troponin	Amperometric	0.1 pg/mL	0.1 pg mL^−1^	11 min	ITO/NC	[91]
Inflammation	Interleukin 6/ C-reactive protein	Amperometric	0.17 pg/mL/ 0.54 pg/mL	0.5–50 pg/mL/ 0.5–500 pg/mL	<20 min	CA/NC	[89]
Prostate cancer	PSA	SWV	0.02 ng/mL	0.05–4 ng/mL	10 min	CA/NC	[101]
Liver cancer	AFP	SWV	0.5 ng/mL	1.0 ng/mL to 100.0 ng/mL	15 min	Gold/NC	[93]
Pregnancy	HCG	ASV	1 mIU/mL	Not reported	13 min	CA/NC	[92]
Cardiovascular disease	MPO	Amperometric	0.18 ng/mL	0.18 and 0.62 ng mL^−1^	5 min	CA/ Celulose	[94]
Dengue	Dengue NS1 protein	EIS	0.5 ng/mL	25 kHz to 0.5 Hz	<5 min	Gold/CF5	[95]
Inflammation	PGE2	EIS	Not reported	100–4000 pg/mL	<5 min	Gold/CF5	[96]
COVID-19	SARS-CoV-2 IgG/IgM	SWV	0.96 ng/mL/ 0.14 ng/mL	1 to 1000 ng/mL	45 min	GO/ Celulose	[97]
COVID-19	SARS-CoV-2	EIS	Not reported	Not reported	15 min	Gold/NC	[98]
Influenza(flu)	Influenza H1N1	EIS	<5 PFU/mL	0–10,000 PFU/mL	6 min	Gold/NC	[99]
Leptospirosis	LipL32	DPV	8.53 pg/mL	0.5–10,000 ng/mL	5 min	Graphene/NC	[102]
Diabetes	Insulin	Chronoamperometry	12 pM	0–250 pM	20 min	CA/NC	[103]
Heart Failure	NT-proBNP	SWASV	750 pM	750–2500 pM	>5 min	CA/plastic	[104]
*E. coli* infection	*E. coli*	DPV	25 UFC/mL	10^2^–10^8^ UFC/mL	10 min	CA/NC	[105]
Inflammation	Interleukin 6/ C-reactive protein	SWV	0.17 pg/mL/ 0.54 pg/mL	0.5–50 pg/mL/ Not reported	20 min	CA/NC	[89]
Liver cancer	AFP	DPV	0.85 ng/mL	0–500 ng/mL	20 min.	CA/NC	[106]
Stress monitoring	cortisol	DPV	10 ng/mL	0–1000 ng/mL	35 min	Graphene/NC	[107]
Inflammation	IL-6/PCT	SWV	0.1 pg/mL/ 0.22 pg/mL	62.5–1000 pg/mL/ 50–800 pg/mL	5 min/ 5 min	Gold/NC	[108]
Prostate cancer	PSA	DPV	0.15 ng/mL	0.01–20 ng/mL	25 min	Graphite/NC	[109]
Biomarkers	ALP/HPV16	Chronoamperometry/ SWV	1.4 U L^−1^/ 1 copy μL^−1^	30–120 U L^−1^/ 1–1000 copies μL^−1^	<1 min/ 2 h	Graphite/NC	[110]
COVID-19	SARS-CoV-2	ECL	7.96 pg/mL	7.9–20,000 pg/mL	5 min	CA/NC	[111]
Smoking exposure	Cotinin	Amperometry	189.7 ng/mL	0–1000 ng/mL	3–60 min	Gold/NC	[112]

LOD: Limit of detection; Ref.: Reference; PSA: prostate-specific antigen; AFP: alpha-fetoprotein; HCG: human chorionic gonadotrophin; MPO: myeloperoxidase; PGE2: prostaglandin E2; DPV: differential pulse voltammetry; SWV: square wave voltammetry; ASV: anodic stripping voltammetry; EIS: electrochemical impedance spectroscopy; CA: Carbon; NC: Nitrocellulose; CY5: Cytiva Fusion 5; SWASV: Square wave anodic stripping voltammetry; ECL: electrochemiluminescence; NT-proBNP: N-terminal prohormone of Brain Natriuretic Peptide; PCT: Procalcitonin; HPV16: Human Papillomavirus type 16; UFC: Colony forming units; PFU: Plaque forming units; IOT: Iridium thin oxide; GO; Graphene oxide.

The integration of electrochemical biosensors and LFPs represents a promising advancement in point-of-care diagnostics, providing significant benefits in terms of analytical sensitivity, quantification capacity, and portability. This technological convergence also offers high sensitivity and wide dynamic detection range of electrochemical systems with the operational simplicity and low cost of traditional lateral flow tests [76,78]. This combination not only improves detection limits and result reliability but also enables real-time quantitative analysis, a limited aspect of efficient colorimetric systems [79,80].

Among the advantages highlighted is the ability of these hybrid devices to operate in complex biological matrices, such as whole blood, saliva, and urine, minimizing optical interference and reducing the need for extensive preprocessing steps [84]. Furthermore, the possibility of multiplexing through the selective functionality of multiple electrodes expands the potential for comprehensive diagnostic panels, suitable for population screening and large-scale monitoring. Protection with emerging technologies, such as low-power wireless communication and artificial intelligence algorithms for advanced signal analysis, points to a future in which these platforms could provide personalized diagnostics integrated into digital health systems [87,88].

However, the integration of these platforms also presents considerable challenges. From a technical perspective, the miniaturization of electrochemical components to the compact format of lateral flow strips requires advances in high-precision microfabrication, the development of optimized conductive materials, and standardization strategies for large-scale production [81,82].

The long-term stability of biological elements immobilized on the electrodes, especially under different storage conditions, remains a significant barrier to the widespread commercialization of these devices [85]. Furthermore, the need for robust clinical validation in various epidemiological and operational settings remains a critical step for transitioning these technologies from the laboratory environment to routine clinical application [85]. From the perspective of specific approaches, each integrated system has its own advantages and limitations.

Enzyme-based systems demonstrate high specificity and signal amplification capacity, enabling analyte detection at extremely low concentrations; however, they rely on controlled conditions to minimize variations in enzyme distribution and performance [78,89,90,91,100]. Systems using nanoparticles offer excellent electrical conductivity and the ability to be functionalized with biomolecules, favoring the amplification of electrochemical signals. However, they can present challenges regarding reproducibility and marker stability under adverse environmental conditions [92,93,94,95,101]. Label-free methodologies, in turn, eliminate the need for additional markers, making assays faster and more economically viable, although they may present lower sensitivity compared to chemical amplification strategies [96,97,98,99].

Thus, while the integration of electrochemical platforms with lateral flow devices offers a promising path toward more sensitive, quantitative, and versatile diagnostics, overcoming the challenges of stability, industrial scalability, and clinical validation will be crucial for their market success. The evolution of this technology will depend on coordinated advances in materials, device engineering, and standardization protocols, as well as the incorporation of complementary technologies that expand its applicability and robustness in real-world usage scenarios.

## 3. Conclusions

The integration of electrochemical biosensors into lateral flow assays represents a significant advancement in point-of-care diagnostics, addressing the limitations of traditional tests, including low sensitivity, poor quantification, and subjective results. This is possible because electrochemical sensors combine the speed of rapid tests with the accuracy of electrochemical analysis, enabling the accurate quantification of complex samples, including clinical, environmental, and food matrices. Furthermore, the possibility of combining functional nanomaterials, such as gold and silver nanoparticles, graphene, and carbon nanotubes, which enhance signal output and aid in receptor immobilization, offers new and additional hope for the development of more accurate tests.

Concurrently, the availability of enzymatic amplification (e.g., HRP and alkaline phosphatase linked to antibodies or aptamers) will enable previously unimaginable levels of detection at femtomolar levels. Another important area of knowledge is electrode surface engineering, which, through the application of chemical modifications, is also contributing to improving target affinity and minimizing background interference.

The incorporation of microelectrodes into lateral flow strips represents another significant advancement in device design, enabling the development of compact, multiplexed systems that simultaneously detect multiple biomarkers, which is often vital for diagnosing complex conditions. Integration with smartphones and real-time software is another factor that could eliminate the need for bulky instruments, facilitating field diagnosis in resource-poor settings.

Advances in portable potentiostats and electrochemical techniques, such as differential pulse voltammetry and impedance spectroscopy, also help maintain analytical performance. Despite progress, several technical hurdles continue to pose difficulties.

Another equally important and concerning point is the scale of production, which requires reproducible protocols, consistent surface chemistry, and stable bioreceptors. The lack of standardization in immobilization and signal reading can compromise the reliability of the results. Systematic clinical validation in diverse populations is also necessary, especially considering biological and environmental variability. While generally cost-effective, integrating nanomaterials into systems can increase the overall cost of the device.

On the other hand, the potential application of these hybrid platforms beyond the healthcare field can justify the investment, with excellent financial returns. In environmental monitoring, they detect pollutants such as pesticides and heavy metals in real time. In food safety, rapid pathogen detection will reduce contamination risks. Wearable biosensors for continuous monitoring of biomarkers, such as glucose and lactate, as well as physical stress, will expand the possibilities in personalized medicine. The future of biosensors is not limited to technological advances, but also to the cooperation and integration of different fields of knowledge. Future progress may depend on the combined effect of AI, 3D printing, and, most importantly, interdisciplinary research. Working together, they can overcome existing barriers and continue to push the boundaries of affordable, fast, and accurate diagnostics.

This work presents a distinguishing feature compared to previously published reviews on separate platforms, as it conducts an integrated and systematic analysis of the union between both systems. It addresses the principal strategies currently employed to overcome the limitations of conventional colorimetric tests in lateral flow systems and those associated with screen-printed electrodes. Although each of these approaches has been independently discussed in literature, they have rarely been explored in conjunction from a comparative and critical perspective. By uniting these technological pillars within a single analytical framework, this study provides a unified overview of ongoing innovations, enabling the identification of complementarities, points of convergence, and persisting gaps. This comparative approach establishes a solid foundation for researchers and developers to understand not only the individual mechanisms of each strategy but also how they can be combined or adapted to generate more robust, sensitive, and accessible hybrid platforms.

## Figures and Tables

**Table 1:** Types of Biosensors and Their Applicability in Clinical Diagnostics.

Diagnostic	Biosensor Type	LOD	Detection Range	Ref.
COVID-19	LSPR Optical fiber-tip	∼0.8 pM (DNA SARS-CoV-2) 2.7 fM (RNA SARS-CoV-2)	m 1 pM to 100 pM	[53]
CVDs	MN	0.67 mg/L	0.5 and 10 mg/L	[57]
*Escherichia coli* 0157:H7 (in food)	SWV	1.65 aM	Not reported	[58]
Glaucoma disease	Amperometric	6.0 pg mL^−1^ (SPP1) 59.0 pg mL^−1^ (GAS6)	20–500 (SPP1) 197–1000 pg mL^−1^ (GAS6)	[59]
CD4 T cells^+^ HIV	EIS	1.41 × 10^5^ cells/mL	1.25 × 10^5^ to 2 × 10^6^ cells/mL	[60]
Thyroid cancer	ECL	0.69 fM	m 1 fM to 1 nM	[61]
Kidney injury	SDI	13.6 aM	10 aM to 1 nM	[62]
Dengue	SPR	310.29°/RIU	Not reported	[63]
Prostate Cancer—PSA	QMC	48 pg/mL	Not reported	[64]

LOD: Limit of detection; Ref.: Reference; HIV: human immunodeficiency virus; PSA: prostate specific antigen; CVDs: cardiovascular diseases; LSPR: localized surface plasmon resonance; MN: microneedle; SWV: square wave voltammetry; EIS: electrochemical impedance spectroscopy; ECL: electrochemiluminescence; SDI: semi-distributed interferometer; SPR: surface plasmon resonance; QMC: quartz crystal microbalance; SPP1: osteopontin; GAS6: growth arrest specific 6.

## Data Availability

No new data were created or analyzed in this study. Data sharing is not applicable.
